# How can we connect with young people? A commentary and recommendations for co-production within qualitative youth mental health research

**DOI:** 10.1177/13591045251364408

**Published:** 2025-08-05

**Authors:** Sophie Dallison, Anastasia Slastikova, Hannah Peel, Grace Chamberlain, Lucy Biddle, Bonnie Teague, Maria Elizabeth Loades, Nina Higson-Sweeney

**Affiliations:** 1 Department of Psychology, 1555 University of Bath, UK; 2 NSFT Research, 8953 Norfolk and Suffolk NHS Foundation Trust, UK; 3 Population Health Sciences, 152331 Bristol Medical School, University of Bristol, UK; 4 Department of Clinical Psychology and Psychological Sciences, University of East Anglia, UK; 5 Department of Experimental Psychology, University of Oxford, UK

**Keywords:** Youth mental health, qualitative, co-researchers, youth involvement, participatory research

## Abstract

Young people (YP) have long been underserved in mental health research. Co-production is part of a significant shift in youth mental health research from tokenistic involvement practices towards more genuine, meaningful collaboration with the group that the research affects: young people. This commentary reflects on learnings from a co-production process in the context of Can We Connect (CWC), a qualitative study on where YP seek mental health information online and their attitudes towards what is available. Young researchers were involved in planning, co-conducting the interviews, contributed to the data analysis and dissemination. Based on our shared experiences as a research team, we aim to provide insights into and recommendations for co-production within qualitative youth mental health research. We (*n* = 12, including 4 young researchers, aged 16–18) reflected on our experiences of either being, or collaborating with, a young researcher in CWC. For us, having young researchers in a research team brings (1) value to YP and (2) value to research. (3) Capitalising on differences is important, (4) having structured support for young researchers and (5) balancing levels of involvement. Open, transparent and honest communication is key to building trust, enabling young researchers to be meaningfully involved members of research teams.

## Plain Language Summary

Young people (YP) have long been underserved in mental health research. Co-production aims to involve genuine, meaningful collaboration with young people, the people who the research affects. This paper reflects on learnings from a co-production process in the context of Can We Connect (CWC), a qualitative study on where YP seek mental health information online and their attitudes towards what is available. Young researchers were involved in planning, co-conducting the interviews, contributed to the data analysis and dissemination. Based on our shared experiences as a research team, we aim to provide insights into and recommendations for co-production within qualitative youth mental health research. We reflected on our experiences of either being, or collaborating with, a young researcher in CWC. For us, having young researchers in a research team brings (1) value to YP and (2) value to research. (3) Capitalising on differences is important, (4) having structured support for young researchers and (5) balancing levels of involvement. Open, transparent and honest communication is key to building trust, enabling young researchers to be meaningfully involved members of research teams.

## Introduction

“No research about us without us” (Michail et al., 2023, p. 1) is an increasingly common expression within mental health research and reflects growing recognition of the need to embed lived experience voices throughout the research process. Patient and Public Involvement (PPI) is often defined as research *with* and *by* patients, rather than *to*, *for* or *about* them (National Institute for Health and Care Research (NIHR), 2021a). In the UK context, but also internationally, PPI and lived experience perspectives are becoming the rule rather than the exception in mental health research (Dewa et al., 2021), with a gradual shift away from tokenistic involvement towards more genuine, meaningful collaboration and integration.

Young people (YP) have historically been considered an underserved group in mental health research (Thomson et al., 2022), yet their involvement in decision-making on issues that directly impact them has been an established right for decades (Conventions on the Rights of the Child, 1989). However, just because it is an established right and increasingly recognised within research guidance, funding and ethics does not mean that YP’s involvement in research is adequately implemented.

Recently, there has been a significant shift towards youth-led research (Michail et al., 2023), whereby YP are considered ‘experts by experience’ and take on the roles of advising for, collaborating on, and leading research about YP (Watson et al., 2023). Research studies that have interviewed YP provide in-depth qualitative insights into on their experiences of research involvement (Dovey-Pearce et al., 2019; Forsyth et al., 2019; Mitchell et al., 2021). The extent to which YP are involved in any research project varies; one particularly popular approach is co-production. Co-production can be defined as a collaborative model of research (Lignou et al., 2019), which extends beyond tokenistic, box-ticking exercises to actively incorporate YP throughout the entire research process. In co-production, YP take an equal and active role, alongside researchers and other stakeholders, in shaping the research, sharing responsibility, and collaboratively generating knowledge (Perowne et al., 2024). The NIHR guidelines on co-produced health research outlined five principles: (1) Sharing power, (2) Including all perspectives and skills, (3) Respecting and valuing the knowledge of everyone, (4) Reciprocity and (5) Building and maintaining relationships (NIHR, 2021b). It is important that co-production in youth research reflects and evidences these principles.

By incorporating YP’s knowledge and lived experience, the research becomes more relevant and meaningful to the end-user of YP. The research benefits, with improvements often seen in data collection, recruitment and translation of findings to practise (Garinger et al., 2016; Pavarini et al., 2019; Warraitch et al., 2024). YP also benefit from an increased sense of empowerment through being given a voice to help others (Michail et al., 2023; Perowne et al., 2024), the opportunity for personal growth, learning new skills and experiencing a sense of community. However, if the research process and environment is ill-supported to meaningfully involve co-production, there is a risk of harm and increased mistrust between researchers and those with lived experience (Williams et al., 2019). As such, it is important for researchers engaging with co-production to continually reflect on and learn from their experiences in order to improve their practices.

## Setting the scene: Can We Connect

**Figure 1.**
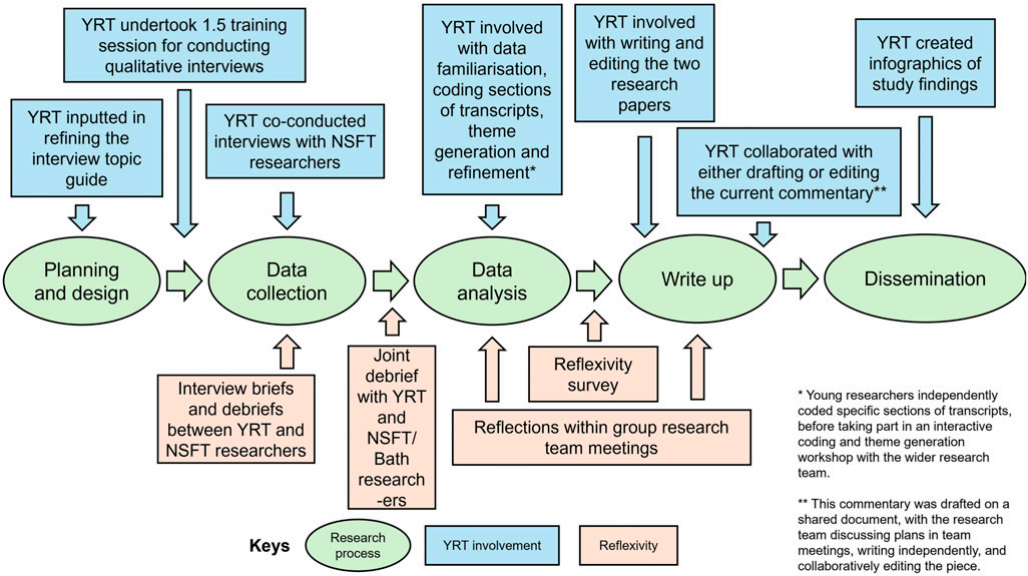
Diagram of the YRT’s involvement in CWC. *Note.* NSFT: Norfolk and Suffolk NHS Foundation Trust; YRT: Young Research Team.

The context for this commentary is the Can We Connect (CWC) study, which was a qualitative study conducted between July-August 2023. UK-based YP were interviewed about where they seek mental health information online, and their attitudes towards what is currently available. The main findings from CWC can be found in Loades et al. (2024) and Higson-Sweeney et al. (2025). However, one of the most interesting elements of the research journey was its underpinning of co-production throughout the phases of the project (see Figure 1 for an overview of their involvement). Specifically, the research team was made up of a combination of academic researchers from the Universities of Bath and Bristol (*n* = 4), clinical researchers from Norfolk and Suffolk NHS Foundation Trust (NSFT, *n* = 5), and a Young Research Team (YRT) comprising four YP aged 16–18 years old with an interest in psychology. The lived experience inclusion criteria to be included as a young researcher was being aged 13–18 years old (see Loades et al. (2024) and Higson-Sweeney et al. (2025) for details of training young researchers received, and the study protocol on the Open Science Framework: https://osf.io/a6k7r). The intent of this collaboration was to utilise our combined skill sets, experiences and perspectives to undertake a high-quality and impactful project, with YP involved as active project partners throughout.

All YRT members received monetary compensation for their time. The YRT were provided with training on how to write for an academic audience and regarding the publication process.

Reflexivity, the critical analysis of the authors’ own position in the research process and how their ‘self-location’ (e.g., gender, class, age, ethnicity) affects their contribution and participation in the production of knowledge (Pillow, 2003), was an important part of CWC (see Figure 1). After data analysis for our first paper (Loades et al., 2024) was complete, two members of the research team (MEL and NH-S) developed a brief open-ended survey for the entire research team to share our thoughts about our involvement of CWC and reflect on the impact of our positionality. The prompts encouraged us to consider i) our identity and how it may have impacted our research involvement; ii) how we engaged in reflexivity throughout CWC; and iii) our experience of either being, or collaborating with, a young researcher. Note that this survey was never intended to be data collection for research, but used as a tool by which we agreed to all share our reflections as part of the research process.

The current commentary showcases some our reflexive discussions and learning as a research team navigating the process of co-production. As a team, including two young researchers who were involved as equal co-producers (step eight of Hart’s Ladder (Hart, 1992)) of this commentary, we worked together to synthesise our experience. The voice throughout this piece reflects everyone’s contributions. Throughout the process of this reflective commentary, we were guided by our research group’s values and mission to meaningfully involve YP throughout research, in a way that is empowering to the young researcher.

By sharing our experiences and reflections, we hope to provide insight into what co-production within the context of youth mental health research might look like, with the overall aim of inspiring future discussions and ultimately contributing to the normalisation of co-production within the field of clinical psychology.

## Reflections

### Value to YP of being researchers

The young researchers amongst us reflected on the numerous ways in which we had benefited from being researchers. Some of us young researchers reflected that co-production provided us with connections with professionals in the clinical psychology field, which initiated networking and the ability to seek advice regarding university applications and career growth. In the context of having struggled to find psychology work experience that was relevant us as under 18s, being part of the CWC research team appealed due to its uniqueness in being research-oriented rather than a generic placement.

As young researchers, we noted the value of the transferable skills we learned, including understanding the process of qualitative interviews, which increased our confidence in communication, analytical skills from coding interviews, and reflexivity. Some of us even felt that being a researcher had enhanced our understanding of our A-Level Psychology courses, particularly the research methods topic, by revealing new aspects of real-life practical research.

Amongst us young researchers, there were differences in the exact benefits that we derived. For one of us, our experience helped to solidify our interest in clinical psychology, whilst for another, CWC involvement opened up ideas about careers in psychological research outside the traditional route.

### Value to the clinical academic researchers of having young researchers

The clinical academic researchers amongst us highlighted the value of having research team members who were the same age as the participants, as we thought that this allowed participants to feel more comfortable and share more. The young researchers amongst us agreed, as we felt we were able to relate to participants’ experiences, having been through the same kind of life events, which potentially created a sense of ease within the interviews. We think this brought a subsequent richness to the interviews that may have otherwise been absent and resulted in data with greater depth.

We think that data analysis was also enhanced by having young researchers involved in the study. The clinical academic researchers amongst us were sensitive to the idea that academia may undervalue the involvement of YP in research, wrongly assuming that without a certain level of training, young researchers would not understand the aims of the research. We were subsequently struck by the advanced levels of understanding and maturity the young researchers brought to CWC, with their insightful and articulate contributions providing a different lens through which the data was viewed.

## Capitalising on differences

Our research team comprised diverse individuals, both personally and professionally. Capitalising on the our different insights was a core part of the process. As highlighted in the previous reflection, age and generational differences within the team were particularly helpful during data collection and analysis, as different questions, topics, answers, and themes were salient to different members, offering greater depth. As CWC was concerned with online help-seeking, these differences were also relevant to our relationships and experiences with technology. Due to being digital natives, the young researchers amongst us had a much more contemporary understanding of social media, including the nuances between different apps and websites, and a better grasp of some of the phraseology used by participants. One of us reflected on how our familiarity with social media as a young researcher meant that we were able to understand its role in influencing YP and their mental health journeys, which was particularly beneficial during the coding and theme generation phases of data analysis.

As a research team, we also had diverse lived experiences with mental health, with different degrees of personal insight allowing the data to be explored from a multitude of perspectives. Equally, some members of the team had experience of delivering mental health treatments to YP, whilst others had (historic) experience of receiving it. Again, this contributed to the depth of analysis and scope of our interpretations. Overall, noticing and taking advantage of our differences strengthened the team’s work and learning.

## Importance of structured support

Going into CWC, there was a clear structure in place for how the young researchers amongst us would be supported by the wider team. This was crucial for both ensuring that ethically and meaningfully conducted co-production remained at the fore and that we as young researchers felt able to fully integrate ourselves within the research process.

As young researchers, we were paired with clinical academic researchers to conduct each interview and were actively involved in leading sections of the interviews (see Loades et al. (2024) and Higson-Sweeney et al. (2025) for details). As a pair, we met for pre-interview briefs and post-interview debriefs. The purpose of briefs was to help us as young researchers grasp the study’s goals and the purpose of the interview, providing us with the necessary background and guidance for meaningful participant engagement. Since the participants were discussing complex and sensitive topics relating to mental health, the briefs were important for helping us feel confident in delivering the interviews. Debriefs were just as important, allowing both members of the interview pair to reflect on and understand our roles in the research. These debriefs gave us as young researchers a chance to assess the quality of the information we had collected, think about how our own biases might have influenced the interviews, and discuss any questions or uncertainties. This kind of reflection was especially important in a study where personal experiences were a large part of the data. As clinical academic researchers, we found this structure equally beneficial.

However, it is important to emphasise that this structured support was underpinned by flexibility and trust. At all times, we endeavoured for there to be a ‘two-way’ relationship between us all as clinical academic and young researchers, where the young researchers felt able to contribute their opinions, perspectives and what they thought could be improved. As young researchers, we valued the openness and adaptability of the clinical academic researchers, which helped us to feel comfortable in voicing their opinions without fear of being ignored or judged.

## Balancing levels of involvement

Some of us as clinical academic researchers reflected on the difficulty of wanting the young researchers to be involved in every part of the process, whilst also being mindful of what they could offer. As a team, we felt strongly about going beyond surface-level involvement and consultation, wanting to ensure genuine co-production. However, as clinical academic researchers working in youth mental health, we were also incredibly aware of the daily demands placed on YP and wanted to avoid adding to them. We therefore took an opt-in approach whereby we invited the young researchers (and also the clinical academic researchers) to be involved in ways that suited them at each stage of the process.

As young researchers, we reflected on feeling valued and enjoyed the level of involvement at each step of the process, giving us a deeper understanding of what was happening and why. The flexibility and adaptability of the CWC study design, and the way the research team worked together, enabled us each to make individual decisions about our level of involvement, helping to mitigate potential overwhelm. We appreciated having the choice to get involved with a range of activities, without the expectation that we would necessarily accept all opportunities offered. For example, we could choose how many interviews to conduct, whether we wanted to code transcripts, and our degree of involvement in writing the research papers. All involvement was digital, which increased flexibility and accessibility.

## What we could have done differently

In thinking together about our shared experiences, we identified areas for future improvement when involving young researchers. This was particularly in the context of qualitative interviews, which is a skill that requires time and practice to develop. As this was the first time that we as young researchers were conducting interviews, we lacked confidence in asking follow-up questions, for example, when the participants said something interesting and deviating from the topic guide. We found that this was particularly the case for the first interviews we co-conducted, but after two or three interviews we were able to relax more and enjoy the process, and we valued the clinical academic researchers’ encouragement as part of this confidence building process. As a team, we feel that this emphasises the importance of structuring the interviews so that they were flexibly co-conducted. This not only meant that the clinical academic researcher could follow up on any missed opportunities for probing participant responses, but also they were there to support us as young researchers, and provide a debrief space for feedback, reflection, and continued growth. On reflection, more roleplay interviews during the training stage, perhaps with a young actor to mimic the interview environment, may have been a helpful addition. Training in data analysis and an introduction to academia upfront could have been useful too, as well as more of an explanation of co-production as a research process. This would have given us as young researchers wider context into how our involvement aligns with co-production.

## Conclusion

This commentary, written by us all as a research team based on our experiences, aimed to normalise and inspire conversations on how to involve YP as researchers when co-producing youth mental health research. Building on the previous literature, we highlight five take-home messages with recommendations for researchers looking to implement a similar process, which have been aligned to the NIHR principles of co-production (NIHR, 2021b) (see Table 1).

**Table 1.** Five take-home messages from CWC.

Name	Message	Recommendation
1. Ensure shared value	Co-produced research can benefit everyone involved; it is not just about what YP can provide to the research, but also what involvement in the research process can offer YP. Walsh et al. (2024) recommend engaging YP in research in ways that enhance their personal development	Implementing co-production in a way that has reciprocal benefits
		• Offering opportunities to shadow researchers for work experience
	NIHR principle: Reciprocity	• Supporting YP to attend conferences
		• Linking YP up with wider networks that may provide further opportunities
2. Provision of support	When involving YP in co-produced research, it is crucial to plan and provide structured support, especially if they have never previously been involved in such projects. Michail et al. (2023) recommended one-to-one debriefs after co-production sessions to highlight what went well and what could be improved	Providing briefs *and* debriefs before and after any involvement with young researchers, to ensure that YP are fully inducted into the research process and allow time for reflection, questions, and continued development
	NIHR principle: Building and maintaining relationships	
3. Authentic involvement	Researchers should attempt to involve YP at every stage of the research process, while being open to taking on feedback and making changes. Hopkin et al.’s (2024) systematic review on the sharing of power in co-production since the NIHR guidelines’ publication highlighted that genuine power sharing is inherently challenging, as different stakeholders may have differing views. Being open to the range of diverse viewpoints of all involved, which may at times conflict, is key to authentic involvement	To allow for authenticity and equitable participation
	NIHR principle: Sharing of power	• Research funders requiring or encouraging co-production need to be realistic about the resources needed for this endeavour
		• Should provide easier access to funded extensions and additional pots of funding
4. Autonomy in involvement	Young researchers should also be given autonomy over what their involvement looks like. Based on our previous experiences and understandings, we may have assumptions regarding how much we should ask of them, for fear of overburdening them	In line with Michail et al. (2023), we recommend
	NIHR principle: Respecting and valuing the knowledge of all those working together on the research	• Clear and open communication with YP about all potential opportunities available to them
		• Clear signposting to the level of commitment involved (e.g., approximate time commitment, reimbursement)
		• Emphasising that there are no expectations regarding how much or how little they want to take on
		• At all times, ensure that YP are in control of their involvement
5. Clearly signpost opportunities	As discussed by Watson et al. (2023), there is a need to increase YP’s awareness of research opportunities	Based on our experience of CWC, we would recommend advertising these opportunities in places that YP are already regularly accessing
		• Social media (remembering to tag organisations that work with YP to share the message)
	NIHR principle: Including all perspectives and skills	• In schools (potentially involving careers services and PSHE leads)
		We also recommend considering how to increase diversity and representation when recruiting young researchers

*Note.* CWC: can we connect; NIHR: National Institute for Health Research; PSHE: personal, social, health and economic education; YP: young people.

In sum, involving YP in co-production represents an important shift in youth mental health research from tokenistic practices towards more genuine, meaningful collaboration. In CWC, we achieved this by involving YP as young researchers who were involved in the entire research process from planning and design through to dissemination. However, this commentary highlighted that the most important element of co-production is transparent and reciprocal communication between YP and researchers, for the mutual benefit of all.
